# Robot-aided assessment of lower extremity functions: a review

**DOI:** 10.1186/s12984-016-0180-3

**Published:** 2016-08-02

**Authors:** Serena Maggioni, Alejandro Melendez-Calderon, Edwin van Asseldonk, Verena Klamroth-Marganska, Lars Lünenburger, Robert Riener, Herman van der Kooij

**Affiliations:** 1 Sensory-Motor Systems (SMS) Lab, Institute of Robotics and Intelligent Systems (IRIS), Department of Health Sciences and Technology (D-HEST), ETH Zürich, Zürich, Switzerland; 2 Hocoma AG, Volketswil, Switzerland; 3 Spinal Cord Injury Center, Balgrist University Hospital, University Zürich, Zürich, Switzerland; 4 Department of Physical Medicine and Rehabilitation, Northwestern University, Chicago, IL USA; 5 Laboratory of Biomechanical Engineering, MIRA Institute for Biomedical Technology and Technical Medicine, University of Twente, Enschede, The Netherlands; 6 Department of Biomechanical Engineering, Delft University of Technology, Delft, The Netherlands

**Keywords:** Assessment, ICF, Robotic rehabilitation, Walking, Muscle force, Range of motion, Proprioception, Synergies, Joint impedance, Gait, Reliability, Validity, Responsiveness, Exoskeleton, Translational research

## Abstract

The assessment of sensorimotor functions is extremely important to understand the health status of a patient and its change over time. Assessments are necessary to plan and adjust the therapy in order to maximize the chances of individual recovery. Nowadays, however, assessments are seldom used in clinical practice due to administrative constraints or to inadequate validity, reliability and responsiveness. In clinical trials, more sensitive and reliable measurement scales could unmask changes in physiological variables that would not be visible with existing clinical scores.

In the last decades robotic devices have become available for neurorehabilitation training in clinical centers. Besides training, robotic devices can overcome some of the limitations in traditional clinical assessments by providing more objective, sensitive, reliable and time-efficient measurements. However, it is necessary to understand the clinical needs to be able to develop novel robot-aided assessment methods that can be integrated in clinical practice.

This paper aims at providing researchers and developers in the field of robotic neurorehabilitation with a comprehensive review of assessment methods for the lower extremities. Among the ICF domains, we included those related to lower extremities sensorimotor functions and walking; for each chapter we present and discuss existing assessments used in routine clinical practice and contrast those to state-of-the-art instrumented and robot-aided technologies. Based on the shortcomings of current assessments, on the identified clinical needs and on the opportunities offered by robotic devices, we propose future directions for research in rehabilitation robotics. The review and recommendations provided in this paper aim to guide the design of the next generation of robot-aided functional assessments, their validation and their translation to clinical practice.

## Background

**Fig. 1:**
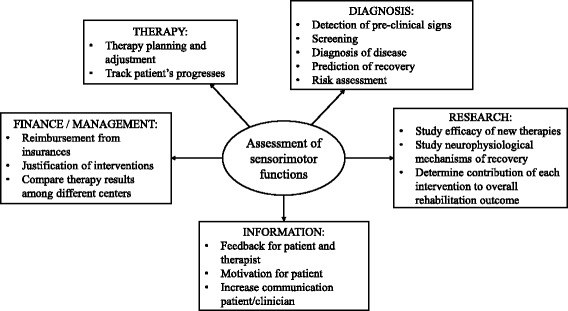
Assessments of sensorimotor functions: purposes. Assessments of sensorimotor functions are needed for several aims [1, 4, 6]: not only assessments are essential in clinical practice to diagnose a disease, to prescribe and to adjust the therapy, but they are also used for management purposes and as feedback for patient and clinician. Lastly, sensitive and reliable assessments are fundamental as outcome measures in clinical trials

Standardized sensorimotor assessments after neurological disorders have the potential to aid the understanding of recovery and to support the design of effective therapeutic interventions, with the ultimate goal of maximizing the patient’s chances of rehabilitation. Despite the general consensus on this statement among clinicians, neuroscientists and rehabilitation engineers, sensorimotor assessments are not routinely performed in the clinical practice [1, 2]. Duncan et al. identified four high-level determinants that impact routine assessments in practice: *i*) Knowledge, Education, and Perceived Value in Outcome Measurement (i.e. information on validity and reliability); *ii*) Support/Priority for Outcome Measure Use (i.e. organizational and management factors); *iii*) Practical Considerations (e.g. time, cost); *iv*) Patient Considerations (e.g. usefulness of the assessment to the patient’s treatment). The limited use of assessments in clinical practice reduces the chances to obtain feedback on the therapeutic intervention and consequently decreases the efficiency of therapy planning and adjustment [1, 3, 4]. Objective proofs are needed to justify healthcare expenses and reimbursement from insurances [1, 3]. In research, the lack of sensitive and reliable outcome measures can hamper the results of clinical trials aimed at determining the efficacy of new treatments, if changes due to the intervention under study fail to be detected [5]. Thus, valid, reliable and sensitive assessments are useful in areas that encompass therapeutic, research and financial domains (Fig. 1).

The last decades have seen an increasing use of robotic devices for neurorehabilitation training in clinical centers [6–8]. Besides training, translational researchers in neurorehabilitation have proposed the use of robotic devices to overcome some of the limitations in traditional clinical assessments. Robotic devices represent an alternative method to provide more objective, sensitive, reliable and time-efficient assessments in clinical practice [6, 9, 10]. Sensors are embedded or can be easily added in robotic devices in order to provide quantitative measures of variables such as, for example, joint angles. Instrumented devices enable the recording of new variables (e.g., smoothness) that were not accessible before. Standardized assessment protocols and repeatable conditions can be achieved with the use of robotic devices. Patient’s motivation, which is a factor that can influence the assessment outcomes [11], can be promoted by using virtual reality applications to provide constant engagement, along with standardized instructions. Moreover, assessments can be integrated into the training session without requiring additional setup and measurement time. Training variables (e.g., duration, number of repetitions) can also be used to provide an indication of the patient’s performance and allow comparison between sessions.

However, the frequent criticism from clinicians towards these engineering solutions is that the outcome measures provided by robotic devices are too abstract, do not translate to function and lack ecological validity. Moreover, robotic devices often require a long setup time and a certain degree of technical knowledge to be operated [6]. In a typical setting, the therapist has between 30 min and 1 h to deliver the therapeutic intervention. If the assessment protocol takes too much time to be performed, the solution may not be adopted. In some cases, the increase in sensitivity and reliability is discarded in favor of an existing subjective, yet time-efficient, assessment that can be applied in any clinical setting. These may be some of the reasons why robot-based assessments have not yet been integrated in clinical practice at a large scale. Therefore, future developments in rehabilitation robotics should enable the clinician to choose among a set of assessment tools according to the specific needs of the patient. We encourage engineers to develop assessment technologies that are not limited by practical constraints and administrative burdens. We believe that the barriers that prevent the translation of robotic assessments to clinical use must be understood so that they can be overtaken. Hence, to guide the development of future robotic-based assessment tools, it is fundamental that we understand the needs of the key players and adjust our motivation to develop new technological solutions.

This paper is targeted to researchers and technical developers in the field of robotic neurorehabilitation. The goal is to provide a comprehensive review of the state-of-art robot-assisted methods, with focus on the lower limb, and identify gaps in which robotic technologies can solve current issues in the assessment of sensorimotor functions. We present and discuss existing assessment methods for lower limb functions used in routine clinical practice and contrast those to state-of-the-art instrumented and robotic technologies. We also provide guidelines and recommendations for the development and validation of new sensor- and robotic-based assessment methods, taking into account the clinical needs. The review and recommendations provided in this paper aim to guide the design of the next generation of robotic devices.

## Framework

Walking recovery is among the most desired goals of patients after a neurological injury [12, 13]. In order to maximize the recovery of the walking function, an optimal therapeutic plan should be defined and adjusted according to the patient’s progress. However, the lack of quantitative and sensitive assessments of lower limb functions that can be used during every day clinical practice limits the possibility to record the patient’s progress over time. For this reason, the scope of this review is constituted by measures and assessment methods that target body functions of the lower limbs, with a particular focus on those related to walking. We decided to exclude assessments of functions that, although needed for walking, are influenced by body systems other than lower limbs (e.g. balance). The methods and papers mentioned in this review were selected from an electronic search in PubMed and Google Scholar. Concerning the robotic measures, for each section we searched for the particular topic (e.g. “range of motion”) and the word “robotic” OR “robot”. Only papers relating to the lower extremities were considered. We looked also at the literature relevant to the robotic gait trainers and exoskeletons. The recent review from Zhang et al. [14] provided a good list of references on ankle devices. We also performed a manual search among the references considered relevant that we found in the selected articles. We aimed at a comprehensive, but not necessarily systematic or exhaustive review.

Assessments of sensorimotor functions can be discussed in the framework of a comprehensive classification for describing health and health-related states developed in 2001 by the World Health Organization. The International Classification of Functioning, Disability and Health (ICF) forms a conceptual basis for the definition, measurement and policy formulations for health and disability [15]. The main aim of the ICF is to provide decision-makers in heath related sectors with a planning and policy tool. Moreover, relevant data can be collected in a consistent and internationally comparable manner. In the ICF, limitations of function and disability are not considered to be etiology-specific but rather are seen as reflecting common manifestations of underlying health conditions [16]. In the same way, the assessments discussed in this review are not disease-specific but are applicable to different kind of populations. The ICF is a useful framework to conceive new robot-based assessment tools and to categorize existing ones. The ICF describes health and health-related states by means of three categories: functioning at the level of body or body part (Body functions and structures), the whole person (Activity), and the whole person in a social context (Participation) [15]. The functions addressed by this review are listed together with their ICF classification in Table 1.

**Table 1:** Lower limb functions and ICF

	Body functions					
Sections of the review	Range of motion	Muscle strength	Proprioception	Joint torque coupling/synergies	Joint impedance	Walking function/Gait pattern
ICF chapters	b710	b730	b260	b760	b735, b7500, b7650	b770, d450
	Mobility of joint functions	Muscle power functions	Proprioceptive functions	Control of voluntary movement functions	Muscle tone functions, Stretch motor reflex, Involuntary contractions of muscles	Gait pattern functions, walking

The sections of the current review in the framework of the ICF. The ICF lists a broad range of health-related components under the categories of Body function (b), Body structures (s), Activities and Participation (d), Environmental factors (e). In each category it is possible to find a complete list of health-related components divided in chapters [211]

## Assessments validation – psychometric properties

In this section, we will present some of the most relevant statistical analyses that are commonly used to evaluate the psychometric properties of an assessment tool. Throughout the paper, we will refer at these definitions to describe the adequacy of the clinical and robotic-based assessment methods. One of the main challenges for the acceptance of new robot-based assessments in clinical practice is their validation. The lack of information on the validity and reliability of an assessment has been identified as one of the barriers to its use [1, 6].

Reliability must be tested first when developing a new assessment method. An instrument cannot be valid if the values it provides from repeated measurements are not consistent [17]. The most common methods to assess the reliability of an instrument in medicine and sport are the Intra-class Correlation Coefficient (ICC) and the Standard Error of Measurement (SEM). The ICC targets the relative reliability (the degree to which individuals maintain their position in a sample over repeated measurements); the SEM measures absolute reliability (the degree to which repeated measurements vary for individuals) [17]. These two methods are, therefore, complementary. ICC values are strongly influenced by the heterogeneity of the subjects (i.e. a high ICC can be obtained even if large differences between trials are present, provided that between-subjects variability is high) [18]. Results of reliability test measured with ICC in a particular population cannot be extended to a study including a different population. The SEM quantifies the precision of individual scores within the subjects [18], but its direct calculation involves the determination of the standard deviation (SD) of a large number of scores from an individual. In practice this is not possible; therefore, the SEM is estimated (Table 2). SEM is independent of the population from which it was determined and it is not affected by between-subjects variability as is the ICC [18]. Absolute reliability can be also evaluated using the Bland-Altman plots [19]. Here, for each subject, the mean of two measurements is plotted against their difference.

**Table 2:** Psychometric properties: Definition and statistical measures

Property	Definition	Measure
Reliability	Consistency of the results obtained on repeated administrations of the same test by the same person (intra-rater or test-retest) or by different people (inter-rater).	ICC: based on ANOVA statistics: between-subjects var/(between-subjects var + error), six different computational methods are possible; 0 ≤ ICC ≤ 1, unitless [212, 213]. Acceptance levels for ICC depends on the application. However, a general classification of reliability has been proposed [214]: 0.00 ≤ ICC ≤ 0.10 – virtually none; 0.11 ≤ ICC ≤ 0.40 – slight; 0.41 ≤ ICC ≤ 0.60 – fair; 0.61 ≤ ICC ≤ 0.80 – moderate; 0.81 ≤ ICC ≤ 1.0 – substantial. $SEM=SD\sqrt{1-ICC}$ (SD of the scores from all subjects). SEM has the same unit of the measured variable [18]. Bland-Altman plots: mean of two measures vs their difference. LOA = ±1.96∙SD [17] Cohen’s Kappa k: percent agreement among raters corrected for chance agreement [215].
Validity	Extent to which the instrument measures what it intends to measure. Concurrent validity: degree to which the measure correlates with a gold standard. Construct validity: ability of a test to measure the underlying concept of interest.	Correlation-based methods: Pearson (r) or Spearman (ρ) correlation coefficient, ICC [216]. For continuous measures of the same data type (e.g. two methods for measuring gait speed): Root Mean Square Error (RMSE) or Bland-Altman plots against gold standard.
Responsiveness	Ability to accurately detect changes. Internal responsiveness: ability of a measure to change over a particular specified time frame. External responsiveness: extent to which changes in a measure over a specified time frame relate to corresponding changes in a gold standard [217] Minimal Detectable Change (MDC): minimal amount of change that is not likely to be due to random variation in measurement [218]. Minimal clinically important difference (MCID): smallest amount of change in an outcome that might be considered important by the patient or clinician [22]. Floor and ceiling effects: the extent to which scores cluster at the bottom or top, respectively, of the scale range.	Internal responsiveness: Cohen’s effect size: observed change in score divided by the SD of baseline score. Standardized response mean (SRM): observed change score divided by SD of change score in the group. External responsiveness: ROC curves: sensitivity vs specificity based on an external criterion [217] $\mathrm{M}\mathrm{D}\mathrm{C} = \mathrm{S}\mathrm{E}\mathrm{M} \times 1.96 \times \sqrt{2}$ [18] MCID: anchor-based (compare a change score with external measure of clinically relevant change) or distribution-based methods (based on statistical characteristics of the sample) [218]. Floor and ceiling effects: percentage of the number of scores clustered at bottom/top.

The presence of systematic bias is confirmed when the mean of the differences between the two tests is significantly different from zero. The limits of agreement (LOA) are another measure of absolute reliability: they indicate the range where, for a new individual from the studied population, the difference between any two tests will lie with a 95 % probability [17]. When the test is used to detect changes between sessions within the same individual, these changes can be considered significant only if they fall outside the LOA. Therefore, the broader the LOA, the larger the minimal detectable change (MDC) would be for a given sample size in an experiment.

Validity assessment is usually more complex because generally the “true” value of a measure is not known with absolute certainty. The general approach for validating robot-based assessments so far consisted in applying correlation between instrumented measures and clinical scores in order to find which parameters measured by robots are able to reconstruct established clinical tests (concurrent validity). However, tying the validation of an instrumented method to a score that is subjective and ordinal-based could be questionable. When a gold standard is already established (e.g. isokinetic dynamometer for muscle strength measurement), concurrent validity can be tested against it. Without such standards, validity is tested indirectly as the ability of a tool to measure the underlying theoretical construct (construct validity) [20].

Responsiveness is the ability of a test to accurately detect change when it has occurred [21]. Reliability highly influences responsiveness because real changes can be masked by the measurement error if the reliability of the test is poor. Measures characterized by a limited number of categories have intrinsically low responsiveness because large changes in status usually are required to the patient in order to change category. Ceiling and floor effects limit responsiveness at the extremes of the score range, since further improvement or deterioration cannot be monitored. The minimal clinically important difference (MCID) is a concept useful to consider the patient’s perspective when dealing with assessments. It involves both a minimal amount of patient reported change and changes important enough to modify patient management [22].

## Overview of clinical assessments and robotic measures of lower limb functions

The following sections provide an overview of assessments methods for different outcome measures. For each outcome measure, its definition and relevance, the ways it is measured in clinic and in research settings are presented. For each of the available instrumented and robotic measures, the advantages over the current clinical assessments as well as points for improvement are also discussed. An overview of the validity, reliability and responsiveness of the clinical assessments discussed in this paper can be found in Table 3. Table 4 provides a list of psychometric properties of available robot-aided assessments. However, the limited amount of studies on validation of the proposed robotic measures prevented the completeness of the table.

**Table 3:** Validity, reliability and responsiveness of clinical assessments of lower limb functions and activities

Measure	Instrument/test	Properties				Study
		Validity	Inter-rater reliability	Intra-rater reliability	Responsiveness	
pROM	Universal goniometer	Knee angle : ICC ≥ 0.98 [219]	Hip flex: 0.56 ≤ ICC ≥ 0.91, SEM = 6.16° [32, 220, 221] Hip ext: 0.20 ≤ ICC ≥ 0.68, SEM = 4.45° [32, 220, 221] Hip abduction: 0.45 ≤ ICC ≥ 0.63, SEM = 6.08°[220, 221] Hip adduction: 0.14 ≤ ICC ≥ 0.65, SEM = 4.4° [220, 221] Knee flex: 0.84 ≤ ICC ≤ 0.93, SEM = 8.21° [32, 220] Knee ext: 0.59 ≤ ICC ≤ 0.86, SEM = 3.48° [32, 220] Ankle DF: 0.26 ≤ ICC ≤ 0.87 [32] Ankle PF: ICC = 0.74 [32]	Knee flex: 0.97 ≤ ICC ≥ 0.99 Knee ext: 0.91 ≤ ICC ≥ 0.98 [222, 223] Hip sagittal angle: 0.51 ≤ ICC ≥ 0.54, SEM = 4° [224] Ankle DF: 0.72 ≤ ICC ≥ 0.89 [34]	-	[32, 34, 219–224]
aROM	Universal goniometer	Knee flex: r ≥ 0.975 Knee ext: r ≥ 0.390	Knee flex: ICC ≥ 0.977 Knee ext: ICC ≥ 0.893	Knee flex: ICC = 0.997 Knee ext: ICC ≥ 0.972	-	[29]
End-feel	Manual examination	-	Hip flex: 0.21 ≤ k ≤ 0.41 Hip ext: k = − 0.13 Knee flex: − 0.01 ≤ k ≤ 0.31 Knee ext: 0.25 ≤ k ≤ 0.43	Knee flex: k = 0.76 Knee ext: k = 1.00	-	[32], [225]
Muscle strength	MMT	Knee flex (vs isokinetic dynamometer): *ρ* = 0.74 Knee ext: *r* = 0.70 [11]	Lower extremities: 0.66 ≤ ICC ≤ 1 [226] MRC score: 0.62 ≤ ICC ≤ 0.88 [227]	Lower extremities: 0.77 ≤ ρ ≤ 0.99 [228]	External resp.: Sensitivity: 60.9 % to 70.3 % [77]	[11, 77, 226–228]
	HHD	Knee ext: 0.43 ≤ r ≤ 0.99 Knee flex: 0.83 ≤ ICC ≤ 0.85 Ankle PF: *r* = 0.93 Ankle DF: *r* = 0.60 [79]	Knee flex: ICC = 0.95 Knee ext: ICC = 0.88 Ankle DF: ICC = 0.69 [78]	Hip: ICC = 0.82 (belt), ICC = 0.80 (therapist) [229] Knee flex: ICC = 0.97 Knee ext: ICC = 0.93 Ankle DF: ICC = 0.91 [78]	95 % CI = 32.5 N (72 %) 95 % CI = 57.1 N (79 %) [229]	[78, 79, 230]
Proprioception	Romberg test	-	-	-	-	
	Toe-test	-	-	-	-	
Joint impedance	MAS	vs ankle measurement device: *r* = 0.09 vs H-reflex: *r* = 0.47 vs Pendulum test: *r* = − 0.69	0.16 ≤ k ≤ 0.61 Ankle PF: *r* = 0.727	0.4 ≤ ICC ≤ 0.75	-	[230]
	Pendulum test	vs MAS: − 0.63 ≤ ρ ≤ −0.89	-	0.651 ≤ ICC ≤ 0.844	-	[153]
Walking function/Gait pattern	WISCI II	Construct validity: vs TUG: *r* = −0.76 vs 10MWT: *r* = −0.68 vs 6MWT: *r* = 0.60	0.98 ≤ ICC ≤ 1	ICC = 1	MDC: 1 level Effect size 2.05, moderate change – discrimination between 1 and 3 months post injury Effect size 0.73, small change – discrimination between 3 and 6 months post injury	[230]
	10MWT	vs TUG: *ρ* = 0.89 vs 6MWT: *ρ* = − 0.95 vs WISCI II: *ρ* = 0.795	*r* = 0.97 LOA = ± 7.0 s	*r* = 0.98 LOA = ± 6.0 s	Effect size: 0.92 - discrimination between 1 and 3 months post injury Effect size: 0.47 - discrimination between 3 and 6 months post injury	[181, 230, 231]

*ρ* indicates Spearman rank correlation, *r* Pearson’s correlation, *k* Cohen’s Kappa, *CI* confidence intervals, *DF* dorsiflexion, *PF* plantarflexion

**Table 4:** Validity, reliability and responsiveness of robot-aided assessments of lower limb functions

Measure	Instrument	Properties				Study and population tested
		Validity	Inter-rater reliability	Intra-rater reliability	Responsiveness	
pROM	Lokomat	-	-	-	-	-
	Isokinetic dynamometer (Biodex System 3 Pro dynamometer - Biodex Medical Systems Inc., Shirley, NY, USA)	-	Ankle DF: ICC ≥ 0.938 SEM = 1.4°	Ankle DF: ICC ≥ 0.930 SEM = 0.8°	MDC = 2.2°-3.3°	[34], 15 stroke patients
	Manual spasticity evaluator	-	*ρ* = 0.95	ICC = 0.86	-	[45], 12 children with CP, 5 able-bodied (AB) adults
	Anklebot	Mean absolute error over two planes ≤1°	-	-	-	[49], validation vs electrogoniometer using a mock-up foot
	Ankle assessment device	-	-	Ankle DF: ICC = 0.846 Ankle PF: ICC = 0.958	Ankle DF: MDC = 3.27° Ankle PF: MDC = 3.81°	[232], 9 AB subjects
aROM	-	-	-	-	-	No studies found
Muscle strength	Isokinetic dynamometer (Biodex System 3)	-	-	Isometric peak torque control subjects: ICC ≥ 0.92; SEM ≤ 25.1 Nm Peak torque patients, contralesional limb ICC ≥ 0.86, SEM ≤ 23.9 Nm	-	[90], 17 subjects with stroke, 13 AB subjects
	Lokomat, isometric test	-	Hip: ICC ≥ 0.87, SEM ≤ 11.2 Nm; Knee: ICC ≥ 0.85, SEM ≤ 7.9 Nm.	Hip: ICC ≥ 0.79, SEM ≤ 10.5 Nm; Knee: ICC ≥ 0.84, SEM ≤ 8.2 Nm.	-	[10], 14 subjects with neurological movement disorders, 16 AB subjects
	Ankle assessment device	-	-	Ankle DF: ICC = 0.949 Ankle PF: ICC = 0.858	Ankle DF: MDC = 1.69 Nm Ankle PF: MDC = 1.68 Nm	[232], 9 AB subjects
Proprioception	Modified Biodex chair, TTDPM test	-	-	Knee frontal plane: ICC ≥ 0.40	-	[104], 17 AB subjects
	Chair with knee actuator, TTDPM test	-	OA: ICC = 0.91, SEM = 2.13°, AB: ICC = 0.89, SEM = 0.43°	OA: ICC = 0.91, SEM = 2.26°, AB: ICC = 0.86, SEM = 0.39°	-	[113] 24 subjects with OA, 26 AB subjects
	Lokomat, JPR test	vs clinical score: Hip: *ρ* = 0.507, Knee: *ρ* = 0.790	-	SCI, Hip: ICC = 0.55, Knee: ICC = 0.882 AB, Hip: ICC = 0.493, Knee: ICC = 0.656	-	[106], 23 SCI and 23 AB subjects
	Lokomat, TTDPM test	vs manual kinesthesia assessment: left hip, r = −0.71; left knee, *r* = −0.86; right hip, *r* = −0.47; right knee, *r* = −0.57	-	AB, hip: ICC = 0.88 left, ICC = 0.94 right; knee ICC = 0.90 left, ICC = 0.91 right. SCI, hip: ICC = 0.97 left, ICC = 0.96 right; knee: ICC = 0.95 left, ICC = 0.96 right	-	[114], 17 SCI and 17 AB subjects Manual kinesthesia assessment: 1 point for each correct movement detection
Abnormal joint synergies	-	-	-	-	-	No studies found
Passive ankle stiffness	Manual spasticity evaluator	-	Ankle DF 4°: *r* = 0.81	Ankle DF 4°: ICC = 0.82	-	[45], 12 children with CP
	Ankle perturbator	Repeated testing of known static torque: ICC = 0.994	ICC = 0.767-0.943	-	-	[233], 10 AB subjects
	Ankle assessment device	-	-	Ankle DF 20°: ICC = 0.863 Ankle DF 30°: ICC = 0.865	Ankle DF 20°: MDC = 0.0686 Nm/° Ankle DF 30°: MDC = 0.1323 Nm/°	[232], 9 AB subjects
Active ankle stiffness	Ankle perturbator	-	-	r > 0.8	-	[164], 11 AB subjects
	Ankle perturbator	-	Between-trial: ICC = 0.76–0.99 and between-day: ICC = 0.64–0.95	-	-	[165], 38 children with CP and 35 AB subjects
Walking function/Gait pattern	Exosuit: strain sensors	Mean absolute error ≤ 8°	-	-	-	[61], 1 AB subject
	Soft ankle orthosis: strain sensors, IMUs	Mean error strain sensor: 0.255 ± 1.63° Mean error IMUs: 0.135 ± 2.85°	-	-	-	[204], 1 AB subject

*ρ* indicates Spearman’s rank correlation, *r* Pearson’s correlation, *DF* dorsiflexion, *PF* plantarflexion

## Range of motion

### Definition of the measure

Range of Motion (ROM) can be defined as the range, measured in degrees, through which a joint can be moved around one of its axes. Active ROM (aROM) is performed by the voluntary movement of the patient, while the assessment of passive ROM (pROM) implies that the therapist (or a robotic devices) rotates the patient’s joint distal segment with respect to the proximal segment [23] while the patient tries to relax. A minimum level of joint ROM is required to perform activities of daily life in a safe and energy-efficient way [24, 25]. For example, reduced knee ROM in the sagittal plane prevents an adequate foot clearance and leads to compensatory mechanisms [26]. After a neurological injury it is common to observe a decreased ROM and a pathological behavior at the extremes of the ROM. To quantify this pathological behavior the “end feel” is sensed, which is defined as the resistance of the joint in response to a gentle overpressure applied at the end of the ROM [23]. A decreased ROM and pathological end feel can be due to weakness, spasticity, pain, tendon and muscle contractures or ectopic bone formation [27, 28].

### Clinical assessment and open issues

The most common instrument used in clinical practice for measuring joint ROM is the universal goniometer. The therapist must place the axis of the instrument over the axis of movement of the joint, aligning the stationary arm with the proximal segment and the moveable arm with the distal segment. pROM is assessed to determine the mobility of a joint regardless of the voluntary ability of the patient and it is usually slightly greater than aROM and much greater in case of muscle weakness. aROM values can be diminished when the movement is performed against gravity, especially in weak patients. When assessing the end-feel, the therapist manually determines the type of this resistance (e.g. “hard”, “soft”, “firm” etc.), which is indicative of different pathologies or conditions that can affect the normal ROM of a joint [23].

Moderate to substantial intra-rater reliability and validity for ROM measurements can be achieved by means of the universal goniometer (Table 3), but inter-rater reliability is generally lower and highly dependent on the therapist’s experience [23, 29–31]. The inter-rater reliability of pROM and of end-feel measurements is particularly critical because it depends on the torque exerted by the therapist on the patient’s joint [30, 32]. Therefore, it is highly recommended that the assessment is performed by the same therapist following a rigid standardized measurement protocol [33]. Additional sources of errors in the measurements are the incorrect identification of the joint axis, the improper alignment of the goniometer arms with the body segments (also due to the movement of the joint) and the parallax error when reading the scale [23]. Moreover, the measures can be affected by compensatory motions occurring at other joints.

### State of the art in rehabilitation robotics

Measures of ROM are obtained through angular position sensors, for which different technologies are available. Within the existing robotic devices available for *clinical use*, isokinetic dynamometers (see section *Muscle force*) embed ROM measurement procedures [34, 35]. Driven gait robots for treadmill walking (e.g. Lokomat [10], LOPES [9], ALEX [36], ARTHuR [37]) and exoskeletons for overground walking (e.g. Vanderbilt [38], Kinesis [39], ReWalk [40], Ekso [41], H2 [42], Vlexo [43]) usually embed potentiometers or encoders in the robotic joints to measure joint angles. Nevertheless, the only method for pROM assessment in a gait trainer available for clinical use is implemented in the Lokomat: the procedure requires the therapist to move the limbs of the patient strapped in the device [44]. For *research purposes*, several attempts to obtain instrumented measurements of the ankle joint have been made, often embedding ROM and stiffness evaluation (see section *Joint impedance*) in the same device. For example, potentiometers were used in two ankle robots to train and assess active and/or passive plantar- and dorsiflexion ROM in stroke patients [45–47] and in a device able to assess ankle rotations in the 3 planes [48]. Another robotic ankle trainer, the Anklebot, embeds encoders to estimate the ankle dorsi-plantarflexion and inversion-eversion angles [49].

End-feel assessment, at the best of our knowledge, has not been realized yet in a lower limb device. Nevertheless, attempts to develop an instrumented end-feel assessment were made for the shoulder joint [50, 51]. The authors used a force sensor to measure the applied force and a motion tracking system to assess the joint displacement. The rationale behind this approach is that the end-feel can be interpreted as the displacement induced by a force applied at the end of the joint ROM. It is, therefore, a measure of stiffness and as such it can be quantified by applying a known force and measuring the joint displacement at the end of the ROM [51]. However, research in this field is still at an early stage and no information on validity and reliability of the measurements are available.

### Future developments in rehabilitation robotics

Rehabilitation and assistive robots usually make use of angular position sensors in their hardware for control purposes and it would, therefore, be natural to conceive robot-aided joint ROM assessments. The development of new technologies in rehabilitation robotics can address many of the issues of current clinical measures of joint motion. aROM measures can be improved by using robots that are able to compensate for gravity while the subject performs active movements, making the assessment independent of the body orientation with respect to gravity. Transparency of robots must be ensured by means of backdrivable actuators or particular control strategies (e.g. admittance control [52], frequency oscillators [53]). The mechanical limits of a robotic joint should be designed in order to allow a subject to reach the whole ROM. Otherwise, the measures will saturate to this limit, leading to an underestimation of the patient’s ROM [9]. The stabilization of the patient’s joints other than the joint of interest and the reduction of compensatory movements can be provided by mechanical fixation to the robotic device. Nevertheless, compensatory movements can be very difficult to detect, especially when they occur within the same joint under test; in this case they can only be identified from the careful eye of the examiner [54]. During the measurement of pROM and end-feel, robots can impose a standardized movement in terms of torque and/or speed [46]. This would improve the reliability of the test making it independent of the operator. Moreover, pre-defined sequences of movements can be programmed using robotic devices in order to have a standardized measurement protocol.

Exoskeletons for overground walking could potentially be used for measurements in static and dynamic conditions provided that gravity, friction and inertia are adequately compensated (see section *Walking function/Gait pattern*). For example, a versatile passive exoskeletal research platform (Vlexo) developed to study human-robot interactions was designed to have robotic joint ROM higher than the human ROM [43]. Each degree of freedom could be blocked to avoid compensatory movement. Thanks to the high adaptability and instrumentation possibilities, it would potentially become a good tool for measuring simultaneously the ROM of hip (abd-adduction, int-ext rotation, flex-extension) and knee in static and dynamic conditions.

End-feel assessment procedures can be implemented with a similar approach as for the shoulder joint [50, 51], using for example motorized exoskeleton devices [55, 56] or ankle robots [46, 48, 49] equipped with angular position and force sensor.

Concerning the measurement technology, the most used angular sensors in robotics are potentiometers, due to their robustness, accuracy and low price. However, since they must be aligned with the joint’s axis of rotation, the measures could potentially suffer from misalignments when the anatomical joint does not have a single axis of rotation or when the setup is not properly done. To overcome this issue, other sensor technologies that do not require the identification of the joint axis can be used. Flexible goniometers based on strain gauge technology are available on the market (e.g. Biometrics Ltd. – uniaxial or biaxial, [57]). The end blocks are fixed to the segments that form the joint and the angle of flexion-extension and abduction-adduction can be recorded, provided that the device is attached in a suitable plane. They have very good performances both in static and dynamic conditions [58–60], but they are at present not sufficiently robust for daily clinical usage. In wearable applications, strain sensors [61] and optic fibers [62] have been used due to their low encumbrance and low weight, but at the moment their performance is not adequate for accurate measurements. Among the wearable sensor technologies, Inertial Measurement Units (IMUs) are promising instruments, given the good performances shown so far, especially in knee dynamic ROM measurements [63–66]. However, they require calibration and signal processing algorithms that perform sensor fusion and compensate for possible inaccuracies due to electromagnetic interferences.

Further studies are recommended to define the hardware configuration, the sensor technology and the measurement protocol that maximize the validity and reliability of the aROM, pROM and end-feel assessment in a clinical context, with the temporal and economic limitations that this implies. Wearable technologies could give an insight of the ROM that the patient is able to display in a real-life situation.

## Muscle strength

### Definition of the measure

Muscle strength is defined as the amount of force generated by muscle contraction [67]. Muscle weakness, or the inability to generate normal levels of force, has clinically been recognized as one of the limiting factors in the motor rehabilitation of patients following stroke [68] and it is one of the major clinical manifestation in hereditary neuromuscular disorders and injuries of the spinal cord [11]. The amount of preserved voluntary muscle contraction has been proven to be highly correlated with walking ability in incomplete SCI [69] and stroke [70]. In the elderly population, lower limb muscle weakness has been associated with an increased risk of falls [71]. In the lower limbs, muscle weakness can be ascribed to disuse atrophy and to the disruption in descending neural pathways leading to inadequate recruitment of motoneuron pools [68, 72]. Assessing muscle strength is important to determine the severity of the injury, to plan the therapy and to monitor the effects of rehabilitation treatments [73].

### Clinical assessment and open issues

In clinical practice, muscle strength is typically assessed using manual muscle testing (MMT) (e.g. Medical Research Council scale [74]). MMT grades strength according to the ability of a muscle to act against gravity or against a resistance applied by an examiner (0: no muscle contraction, 5: holds test position against maximal resistance) [73]. However, the accuracy and sensitivity of MMT is low and the same grade in MMT corresponds to a large range of absolute strength values [73]. It was reported by [73] that Beasley found that a variation of less than 25 % in muscle strength for the knee extensor cannot be detected by MMT [75]. MMT is strongly influenced by the experience of the examiner, who must avoid compensatory movements by the subject and ensure a standard positioning. MMT suffers from ceiling effects, because the maximum score (5.0) is assigned before a normal level of muscle strength is truly reached [76]. MMT was found not adequate as a screening tool and insufficient in tracking the progress of a patient undergoing therapy [77, 78]. Subtle increases in muscle strength are only detectable with instrumented methods.

Quantitative measures of muscle strength can be performed during isometric, isoinertial or isokinetic contraction. In an isometric test the subject is asked to perform a maximum voluntary contraction (MVC) against a fixed resistance and the maximum value of the force/torque is retained. In clinical practice, this test is mostly performed with a hand-held dynamometer (HHD) or myometer. The HHD is a portable force sensing device that can be placed between the hand of the examiner and the body segment to test, similar to how an examiner would perform a MMT [79]. The examiner must be able to apply a resistance equal or greater than the patient’s force. Like the MMT, the myometry is, therefore, depending on the amount of resistant force the practitioner is able to apply to the segment of interest and on his ability to stabilize proximal joints [79, 80]. Nevertheless, with respect to MMT, myometry has higher sensitivity and it is less prone to ceiling effects [73]. Reliability and validity of HHD measures can be further increased by fixating the device with a belt [81, 82], so that the resistance applied against the movement is not dependent on the examiner’s force. Load cells mounted on supportive frames can also be used for this purpose [83]. Isoinertial tests consist in lifting a constant load throughout the joint ROM and the outcome is the maximum load that can be lifted once (1-RM) [84]. Isoinertial tests are usually executed using sport devices, like the leg extension machine, modified in order to record the joint angle [85]. During an isokinetic contraction the joint angular velocity is kept constant by a machine, the isokinetic dynamometer. The subject is asked to forcefully contract the muscles during the whole ROM while the peak torque is calculated. This test can only be performed with a robotic device and it will be discussed in the next section. Isokinetic tests could be useful to unmask speed-dependent strength impairments [86]. Although the isokinetic dynamometer is considered the gold standard for muscle strength measurements, price, encumbrance and setup time limit its use in a clinical setting. Therefore, it was proposed to use preferably isometric or isoinertial tests in clinical practice due to their reduced cost and easiness of use [84, 85]. The three test modalities have indeed similar good construct validity (relation with physical function) and substantial test-retest reliability [85] and high correlations have been found between isometric and isokinetic torque measures, although isometric tests lead normally to higher values of muscle strength [84, 87]. It is important that users are aware that these three conditions provide different estimates of muscle strength. Nevertheless, it was demonstrated that using the HHD according to standard procedures and fixation, excellent inter and intra-tester reliability and a good correlation with the isokinetic dynamometer can be achieved [73, 79, 81, 88]. Therefore, given the cost and long measurement time (around 25 min) required by the isokinetic dynamometer, it was suggested to favor the use of HHD in clinical practice [79, 88].

### State of the art in rehabilitation robotics

The most known device for muscle strength measures is the isokinetic dynamometer. This machine allows the measurement of joint torques in controlled conditions: isometric at selected joint angles or isokinetic at selected angular velocities [79, 89]. A servo-controlled lever arm provides resistance to the subject’s joint when it reaches a defined angular velocity (≥0 deg/s). Different mechanical configurations allow testing of hip flexion-extension and ab-adduction, knee flexion-extension, ankle plantar-dorsiflexion and eversion-inversion. The patient’s trunk and the segments proximal to the joint tested must be stabilized with straps and the axis of the dynamometer must be carefully aligned with the axis of the joint to test to avoid measurement inaccuracy [89]. In isokinetic tests the subject is asked to push “as hard and as fast as possible” while the device provides resistance to the movement of the limb so that it cannot accelerate beyond the machine’s preset angular speed [90]. A continuous passive motion (CPM) has been proposed for severely impaired subjects, where the robot moves the limb and the dynamometer lever arm at a preset velocity while recording forces applied to the lever arm [11]. Reliability and validity of the isokinetic dynamometer are substantial but the high cost and the long setup time limit its use in everyday clinical practice.

In rehabilitation robotics, muscle strength has been measured integrating force sensors into the structure of exoskeletal devices for quantifying physical human-robot interaction and estimating the force exerted by the patient. Directly measuring the interaction force at the attachment points requires a load cell, placed at the connection between the cuff/orthosis and the exoskeleton link, such as in a modified version of the Lokomat [91, 92]. Otherwise, the estimation of interaction torques can be achieved through a force sensor in series with the actuators, like in the Lokomat [44] and in the ALEX [36], or through linear potentiometers for measuring the length of the springs used in the actuators of the LOPES I [93]. The torques produced at each joint are calculated online from the joint position and the linear force values. The Lokomat, in particular, allows the execution of hip and knee isometric strength tests in the sagittal plane: the patient is positioned with 30° hip flexion and 45° knee flexion and asked to flex or extend the joints against the resistance provided by the orthosis. A moderate to substantial inter- and intra-rater reliability of this method was found with patients with and without neuro-muscular disorders [10].

The ankle joint is usually measured separately from the hip and knee joints with dedicated devices used in a sitting position [14, 94]. An ankle robot constituted by a footplate fixed through a six-axis force sensor to a servomotor shaft that controls its angular position and speed was used for measuring isometric muscle strength: the subject’s ankle was locked at the 0° ankle dorsiflexion, and maximal voluntary contraction was taken [46, 47]. Isometric torques of the ankle joint in different kinematic configurations were obtained from a device able to measure ankle torques around the three articular axes (plantar-/dorsiflexion, int-/external rotation and pronation/supination). The 6-DOF structure allows linear and angular displacement of the ankle with respect to the shank. Each DOF is blockable in different configurations and torques and angles can be measured [48].

### Future developments in rehabilitation robotics

Despite the poor psychometric properties of the MMT, methods alternative to this test that can be easily integrated in a clinical setting are lacking. Robotic devices can address many of the problematics previously identified. The responsiveness of muscle strength tests is important for detecting small changes during the progression of rehabilitation. Therapy goals can be set based on the minimum force required for performing activities of daily living, like walking or sit-to-stand [95, 96]. It is important that a test is able to detect changes at least equal to the MCID. However, MCID of muscle strength changes in patients with neurological disorders have not yet been established. Ceiling effects must be avoided in order to have a measurement scale that can be used also with mildly affected patients. Robotic devices have the potential to provide more sensitive assessments thanks to the sensors embedded in their structure. Standard and repeatable testing conditions can be achieved by implementing a system for fixating the patient to the device and preventing undesired movements and by programming a standard sequence of movements that should avoid fatigue effects [97]. Moreover, assessment procedures can be integrated in a therapy session performed with a rehabilitation device without requiring additional setup time.

The isokinetic dynamometer is a first attempt to provide a state-of-the-art robotic assessment method [98]. A large body of research on this device have unraveled the possible shortcomings and studied different applications and measurement protocols. In particular, factors such as gravity compensation, damping of the system, human-machine interface and alignment of the human and robot axes of rotation have been considered in many publications [85, 89, 99]. This knowledge can be applied to the development of future robot-aided muscle strength assessments, despite the fact that the differences in hardware prevent the complete reproducibility of the results. Testing subjects with severe weakness requires particular attention because subtle levels of muscle strengths can be masked by the use of device that is too heavy for the patient or the use of a position that does not eliminate the effect of gravity [11]. Lastly, the motivation of the patient plays an important role [11] and it would be worthy to investigate how this human factor affects the outcome measures and consequently to standardize the protocol and the instructions.

## Proprioception

### Definition of the measure

Proprioception can be defined as the ability of an individual to determine joint and body movement (kinaesthesia) as well as position (statesthesia) of the body, or body segments, in space [100, 101]. It is based on sensory signals provided to the brain from muscle, joint, and skin receptors [102], with muscle spindles playing the major role [103]. Proprioceptive feedback has been demonstrated to be a key component of motor control and functional joint stability [104]. A diminished proprioceptive acuity at the ankle joint is associated with a lower unipedal stance time, which is a measure relevant for evaluating frontal plane postural control [105]. Loss of proprioception has been reported both in neurological (e.g. stroke, SCI, peripheral neuropathy) and in orthopedic patients (e.g. knee osteoarthritis) and it has been associated with an increased risk of falls in the elderly [103].

### Clinical assessment and open issues

Assessment of lower limb proprioception in clinical practice is based mainly on two rather simple tests: the movement detection at the big toe and the Romberg sign [103]. In the first the examiner moves the patient’s toe upward or downward and asks to detect the direction and the amplitude of the movement. In the Romberg test, the subject is asked to close his eyes while standing with his feet close together. A non-specific proprioceptive deficit would usually result in the loss of balance. While useful as a quick method to detect the presence of proprioceptive abnormalities, these tests are not sensitive enough to detect mild impairments or to track changes over time. Moreover, the test at the big toe depends strongly on the pressure applied by the examiner and the amplitude of the movement imposed [106]. Furthermore, only the distal segments of the upper and lower limb are tested and no assessments of the proximal joints are performed. A more specific test, even if less used in clinical practice and mainly in upper limb examination, is the joint position reproduction or matching (JPR) [100]. In this test the patient is blindfolded and the examiner moves his/her limb to a target position. The patient is then asked to match this position either with the contralateral limb or with the same limb after it has been brought back to the starting position. This test is normally performed without any instrument and the visually observed mismatch in position is retained as a rough measure of proprioceptive precision [102, 107]. Goniometers can also be used to measure the joint angle before and after the matching but their reliability and measurement error have been shown to vary widely [108]. Items related to proprioception are included also in the sensory-related section of the Fugl-Meyer Score for stroke patients. Here small alterations in the position of hip, knee, ankle and great toe are evaluated [109]. However, the stimulus provided by the examiner is inherently subjective and sensitivity is limited to 3 levels (absent, impaired or normal proprioception).

### State of the art in rehabilitation robotics

Instrumented tests for proprioception in lower limbs have been developed using motorized devices or isokinetic dynamometers. An overview of these experimental devices and methods can be found in [100, 110].

The classic JPR test discussed above can be easily instrumented. A machine moves the subject’s limb to the target position. The subject is then asked to match this position, either by actively moving the limb or by pressing a button when the limb passively moved by the machine reaches the target position. However, it has to be taken into account that active and passive motion of the limbs are not equal in terms of sensory feedback [107]. JPR methods are not suitable for people with cognitive impairments since they are highly dependent on memory [100]. Moreover, they have been found to have slight to moderate reliability [107]. A JPR test for assessing hip and knee joint proprioception has been implemented in the robotic gait orthosis Lokomat and tested in healthy subjects and 23 incomplete SCI subjects [106]. The subject’s leg was positioned at a target hip and knee angle and then moved away to a distractor position. The subject was then asked to place the limb at the remembered target position using a joystick to control the robot. The absolute error between target and remembered position was retained as outcome measure. The test-retest reliability in SCI was found to be fair at hip joint and substantial at the knee joint but the Bland-Altman plots showed broad LOA that indicate a low sensitivity in SCI individuals. Heteroscedasticity was also reported. Nevertheless, the score correlated well with the clinical assessment of proprioception and a significant difference between SCI patients and healthy subjects was found.

A second approach for measuring proprioception is the threshold to detection of passive motion (TTDPM). In this test the body segment under test is moved by a machine in a predefined direction. The subject is asked to press a button as soon as he/she detects a movement. Movements are presented at different velocities since the proprioceptive threshold decreases with increasing speed [100, 111]. A motorized apparatus for testing hip, knee, ankle and toe detection threshold was developed by Refshauge et al. and the influence of speed and joint position on the test outcomes was studied [111, 112]. A modified isokinetic dynamometer and a chair with motorized arms have been used for assessing passive flexion/extension and varus/valgus movements of the knee in healthy subjects and osteoarthritic patients (OA) [104, 113]. From the initial posture, the servomotor rotated the knee at a constant low velocity of below or equal to 1°/s). The threshold position of detection of the movement was retained, with smaller threshold values indicating greater proprioceptive acuity. Reliability was found to be excellent both within and between raters, both for OA and healthy subjects. In both studies the subjects wore headphones and an eye mask. The TTDPM was tested also using the Lokomat [114]: hip and knee separately were moved according to a randomized order of speeds (0.5–4°/s), directions and catch trials (no movement). Angle and reaction time were used to calculate a movement detection score. The score presented substantial reliability and a high correlation with a clinical score of proprioception, showing better sensitivity (it is possible to measure reaction times ≥ 50 ms) and no ceiling effects. Faster speeds were able to elicit a response in severely impaired subjects that could not detect movements at 0.5 °/s. The TTDPM test leads generally to more precise and less variable measures of proprioception acuity than the JPR test. Interestingly, the two tests have shown no concurrent validity [107].

### Future developments in rehabilitation robotics

These studies demonstrate that instrumented and robotic assessments of proprioception are feasible and present several advantages over clinical assessments of proprioception. Measures of proprioception in clinical practice are rather coarse and lack granularity. Standardization is nearly absent and the outcome of clinical tests is often a binary answer.

Lower limb robotic devices provide the possibility to maintain a high consistency in the protocol (speed, points of contact, timing) between trials. The responsiveness of the robot-based measure was demonstrated also by the ability to detect a wide range of angle errors in subjects that were judged unimpaired by the clinical assessment [106, 114]. Moreover, the influence of motor impairment on the control of lower limbs can be eliminated because the leg can be passively moved by the robot. Lastly, robotic devices can provide useful information on joints that are not normally addressed in clinical practice, where the most common examination involves only the big toe [103]. It is likely that specific information on other joints might provide an insight on different components of sensory function useful to track changes in recovery after injury [114]. On the other side, the straps of exoskeletal devices may provide additional cutaneous feedback to the subject, thus influencing the measurements [114]. When designing a new robotic device or protocol for proprioception assessment it is important to consider that the test methods (JPR or TTDPM) do not provide the same information [107]. Different versions of the protocol exist also within the same test and again their choice can highly influence the results [102]. The speed of a TTDPM test highly influences the outcome measures [100, 114] and must be accurately controlled by the robotic device. Active and passive movements are likely to activate different proprioceptive mechanisms [107].

Robot-based assessments of proprioception require longer time of administration with respect to clinical assessments, but they are able to provide reliable and sensitive information on proprioceptive acuity that allows a more detailed examination useful for diagnosis or accurate tracking of the recovery of the patient.

## Abnormal joint torque coupling and synergies

### Definition of the measure

Due to cortical damage, stroke survivors and cerebral palsy (CP) children can lose the ability to move their joints independently, which result in abnormal coupled, pathophysiological movement patterns, also called synergies. The loss of independent control of joint moments is caused by involuntary co-activation of muscles over multiple joints [92].

Brunnstrom [115] defined two often occurring pathophysiological synergies in the lower extremities:

1. Extension synergy consisting of internal rotation, adduction and extension of the hip, extension of the knee, and plantar flexion and inversion of the ankle
2. Flexion synergy consisting of external rotation, abduction, and flexion of the hip, flexion of the knee and dorsal flexion and eversion of the ankle

### Clinical assessment and open issues

Loss of independent joint control limits the performance on activities of daily living. Therefore, in both clinical and in research settings abnormal joint torque coupling is often being assessed and this is mostly done using the Fugl-Meyer Assessment of Physical Performance [116]. This scale has been shown to be a reliable, sensitive and valid method for the assessment of motor impairment after stroke [117–119]. However, it can be argued that for the quantification of abnormal joint torque coupling this scale lacks sensitivity due to the use of a 3-point scale (0 = cannot perform,1 = performs partially, 2 = performs fully) for the assessment of each component of torque coupling.

### State of the art in rehabilitation robotics

Robotic and robot-related measures could possibly provide more accurate information. Over the last decade several studies have investigated abnormal joint torque coupling using robotic and robot-related measures [68, 92, 120–126]. The majority of these studies quantified the synergies in static situations during isometric contractions and used a similar approach. Subjects were strapped into a (robotic) device (most often the Lokomat) that constrains every movement of the concerned leg and the pelvis. The device was equipped with force sensors to measure all the interaction forces/torques that the subject exerts with this leg on the device, for instance the cuffs of the Lokomat were instrumented with 6-DOF load cells [92, 120, 121]. Participants produced isometric torques in a particular direction (primary), while torques in all other the directions (secondary) were also measured. Abnormal torque coupling was quantified as the difference in secondary torque production between healthy individuals and stroke survivors. Studies differed in the amount of joints and planes that were investigated and the position in which the coupling was assessed. Thelen et al. [123] assessed the coupling while subjects were positioned in an adjustable chair with ankle fixed to six degree- of-freedom load cell, whereas others assessed the coupling while subject where standing in the toe-off and/or mid-swing position with the test leg unloaded [68, 120–122].

Thelen et al. showed that individuals with cerebral palsy produced a knee extension moment during hip extension and vice versa whereas healthy subjects produced a knee flexion moment during hip extension and a hip flexion moment during knee extension. Quantification of abnormal joint couplings using a (robotic) device has provided evidence for different couplings. Neckel et al. [68] found that stroke survivors only showed an abnormal coupling between hip abduction and flexion and had similar couplings as found in healthy subjects for the other degrees of freedom. Cruz and Dhaher [121] observed that stroke survivor coupled knee extension with hip adduction. Tan et al. [120] found strong coupling between ankle frontal plane torque and hip sagittal plane torques and vice versa that were not present in the healthy control subjects (ankle plantar flexion with hip adduction, ankle eversion with hip extension and ankle inversion with hip flexion). Recently, Sanchez et al. [127] also found evidence for the earlier found coupling between hip extension and adduction, and ankle plantar flexion and hip adduction. So, evidence starts to accumulate that stroke survivors have abnormal coupling between hip adduction, hip extension and plantar flexion.

To our knowledge only one study has attempted to identify abnormal joint torque coupling during walking [92]. In this study participants were moved along a predetermined locomotor trajectory using the Lokomat while interaction and ground reaction forces were measured. However, the difficulty with this setup is that it is hard to disentangle the torques required for walking and maintaining balance and those resulting from the abnormal joint torque coupling. Therefore, although assessed in a quasi-dynamic situation, the results may not be generalizable to voluntary walking.

The reliability (test-retest, inter-rater, intra-rater) has not yet been assessed for these abnormal couplings, nor has its responsiveness been determined. The criterion validity has not explicitly been investigated, however Cruz and colleagues [128] demonstrated using step wise regression that the coupling between knee extension and hip adduction was the best predictor of gait speed amongst other strength and coupling variables. None of the aforementioned studies did correlate their coupling measures with a clinical scale like the Fugl-Meyer to assess the construct validity.

### Future developments in rehabilitation robotics

To summarize, robotic measures may be able to quantify abnormal joint torque coupling more precisely compared to clinical measures such as the Fugl-Meyer Assessment of Physical Performance. However, the reliability, responsiveness and validity of these measures need to be further investigated. Additionally, robotic assessment is still performed under static or quasi-dynamic conditions, which might not quantify well how these couplings limit walking. For assessing abnormal couplings in the upper extremities, the assessments have moved from a static approach [129] to a dynamic approach where the couplings are assessed during reaching movements using robotic devices [130]. We foresee that a similar shift will happen for the lower extremities. Integration of the principle used in the robotic assessment under static conditions in robotic gait trainers could provide the tools to assess abnormal joint torque coupling during walking.

## Joint impedance

### Definition of the measure

In the clinical field, the term *joint stiffness* has been used to express the sensation of difficulty in moving a joint [131]. While this term is commonly used in the clinical practice, the notion of *stiffness* used in this context does not match the definition of stiffness in classical mechanics. To describe all the mechanisms that contribute to the resistance of motion, the term *impedance* is usually preferred. In motor control literature, the term mechanical impedance is defined as the dynamic operator that specifies the force an object generates in response to an imposed motion [132]. The latter definition includes all motion-dependent effects, i.e. those terms that specify the force generated by changes in position (e.g. stiffness, non-elastic forces), in velocity (e.g. viscosity, damping) and in acceleration (e.g. inertia) [133]. In biomechanics, the term *joint impedance* relates the motion of the joint and the torque acting about it [134]. Joint impedance is usually estimated by applying a torque or force perturbation and measuring the resulting change in position or applying a position perturbation and measuring the resulting change in torque of force.

Joint impedance is mainly determined by three sources: *i*) the passive biomechanical properties of the muscles, tendons and tissue around the joint and limb inertia – *passive* components; *ii*) the resistance produced by the muscles in response to reflexes [134–137] – *reflexive* components; and iii) the resistance produced by the muscle fibers due to non-reflexive, neural-driven contractions – *intrinsic* components [137]. Since the *reflexive* and *intrinsic* component are both related to muscle activation, their sum is commonly referred to as *active* component^1^.

In neurological populations, an abnormal increase in joint impedance can result from spasticity, rigidity or dystonia [138]. The intrinsic and reflexive components have also been shown to be affected in neurological populations [139].

Joint impedance varies with muscle contraction [140], joint position [141–143], rotation amplitude [144], and the duration of the applied perturbation, since after approximately 30 ms cross-bridges break [145] and the contribution of cross bridge stiffness to the overall joint impedance will diminish. Joint position affects joint impedance measurements because the intrinsic component increases towards the extreme joint angles as the ligaments get more stretched. Additionally the different muscles vary their active contribution to the joint impedance depending on their length (and therefore on the corresponding joint configuration), due to the particular shape of the length-tension curve of the muscle [146]. The reflex activity is also known to be speed dependent [147] and only contributes above a threshold [148]. Finally, the task instruction given to the subject will also shape the joint impedance [149]. Most common task instructions are ‘relax’,’resist the perturbation’, or ‘keep the force constant’.

### Clinical assessment and open issues

The Modified Ashworth Scale [150] is the most widely used clinical assessment to quantify an abnormal increase in joint impedance due to excessive muscle tone. The MAS consists of moving the limb of the patient through its range of motion and rating the resistance on a 6-point scale. The MAS is widely accepted, even though the validity and reliability of the measure are questionable [151] since especially inter-rater reliability was slight to fair. Moreover, the MAS may also lack sensitivity. The MAS assess joint impedance only in passive conditions, where the subject is asked to relax, which might not be indicative for how spasticity influences dynamic movements. Another test to assess the increased resistance to movement in a more quantitative way is the pendulum test, first described by Wartenberg [152]. This test quantifies movements of the lower leg following its drop from a horizontal position by deriving the angle of first reversal, the maximal angular velocity or number of oscillations. The pendulum test has shown good convergent validity, reliability and sensitivity [153, 154]. Some limitations of this test are that it is done in relaxed conditions – which is difficult to achieve - and can only be used for the knee. Additionally, measuring equipment (electrogoniometers, inertial sensors) are needed to record the leg motion and to extract the variables.

While measurement of joint impedance in not commonly performed on the everyday clinical practice, it has implications in understanding a potential cause of impairment. For instance, Mirbagheri et al. [139] was able to isolate abnormal active contributions in spinal cord injury patients based on measurement of joint impedance of the ankle. Such measurements can also point out to different pathologies such as spasticity, rigidity or dystonia [138].

### State of the art in rehabilitation robotics

As mentioned earlier, joint impedance is dependent on joint position, muscle contraction levels, and amplitude, velocity and duration of the perturbation. Therefore, the use of robotic devices is advantageous because these factors can be precisely controlled at the same time relevant signals are been recorded. Several instrumented and robotic measures have been developed to asses either the reflexive and/or intrinsic components of joint impedance [45, 49, 136, 138, 155–162]. We will not review all devices and methods. In particular for the ankle joint many devices have been developed, which have recently been reviewed [14]. To assess passive joint impedance, the joint of the participant is moved by a robotic manipulator or manually over a certain angle often measured using a potentiometer while the resisting force is measured using force sensors integrated in the (robotic) device. For accessing the passive joint impedance it is important that no muscle activity is present. Therefore, the participant is asked to (try to) relax and the angular velocity is kept low to avoid the excitation of reflex contractions. In the push and pull test, the joint is moved with small increments and kept static for approximately 5 s in every position. The net moment (after removing gravity) provided by an external device to keep the segment in equilibrium is retained for each incremental position [163]. Both isokinetic dynamometers and custom made joint actuators have been used as assessment devices. With a manually operated device the passive ankle impedance could be estimated reliably in healthy subjects (ICC values between 0.71 and 0.85,[156]) and in CP children (ICC = 0.82, [45]). In the study of Chesworth et al. [158] a custom made torque motor system was used to assess passive joint impedance of the ankle with a comparable reliability (ICC: 0.77–0.94).

The contribution of active components (i.e. intrinsic and reflexive) to joint impedance have also been investigated using similar experimental setups. In a typical setup, the subject is either asked to actively resist an angular displacement or to (try to) exert a constant force. At some point, either an angular position perturbation is applied while the resisting force is measured or a force perturbation is applied and the resulting angle is measured. Impedance measured under this condition contains the three components: passive, intrinsic and reflexive. To be able to distinguish between these components, different strategies have been used. For example, in the study of McHugh et al. [160] the passive component is subtracted from the total impedance to determine the active component. Also more complex methods exist, which are based on system identification techniques. In the method of Mirbagheri et al. [136], a system identification method is applied to distinguish between intrinsic and reflexive components. In this method, pseudo-random continuous rotations of the ankle are applied, and the ankle torque and EMG of involved muscle groups are recorded. The model consists of an intrinsic component and a unidirectional delayed velocity feedback pathway representing the reflexive component. Input to the model is ankle rotation, and the model parameters are optimized to minimize the error between the predicted and recorded torque. The EMG is used to determine the latency of the reflex component. In healthy subjects a good intra-rater (r > 0.8) reliability was found [164]. De Vlugt et al. [162] used similar techniques but instead of continuous rotations, they applied ramp-and-hold ankle dorsiflexion rotations with different speed profiles. They employed a nonlinear neuromuscular model that is more complex than the one used by Mirbagheri to predict the recorded ankle torque. Results showed that stroke survivors could be distinguished from control subjects by tissue stiffness and viscosity and to a lesser extent by reflexive torque from the soleus muscle. These parameters were also sensitive to discriminate different patients, who were clinically graded by the MAS. In a subsequent study [165] these researchers adapted their model and protocol slightly by applying both ankle plantar and dorsiflexion rotations. The estimated model parameters could discriminate between patients with CP and control subjects. Soleus background activity was sensitive to MAS spasticity severity, but reflex activity was not. Preliminary data indicated that reflex activity was reduced after spasticity treatment. The between-trial (ICC: 0.76–0.99) and between-day repeatability (ICC: 0.64–0.95) was moderate to substantial for tissue stiffness and background activity, but not for reflex parameters.

A shortcoming of most of the studies on joint impedance is that the assessment is done for static or passive tasks where the participants are in a supine, prone-lying or sitting position. The ankle impedance has also been determined in more natural active conditions, such as stance [166, 167] using very fast dorsi- and plantar-flexion rotations with a motorized footplate and non-parametric impedance estimates.

Aforementioned studies and approaches all made use of dedicated assessment setups. However also robotic gait trainers can be used to derive measures of joint impedance. For instance, the Lokomat has a built-in function to assess overall joint impedance of the hip and knee joints by passively moving the limbs at different speed profiles and recording the resulting joint torque. Using this technique, a moderate correlation between joint impedance and MAS scores could be seen [157]. Koopman et al. [168] used the LOPES robotic gait trainer and multi input multi output system identification techniques to assess joint impedance of the hip and knee. Healthy subjects were assessed while in the toe-off or heel strike position and were asked to resist the movement of the device or apply no force at all. Results showed that the effect of biarticular muscles on the inter-joint impedance could not be ignored.

### Future developments in rehabilitation robotics

Although research on the accuracy and reliability of robotic devices to assess joint impedance is not available for all developed devices and methods, it can be argued that the use of integrated sensors and robotic actuators will show better psychometric properties compared to the MAS score. Another advantage of robotic measures is that they can help to develop methods to estimate the active and passive or intrinsic from reflexive components, while the MAS only measures the resistance of motion but not the underlying cause. The pendulum test could be implemented in combination with transparent devices that do not hinder the natural oscillation of the shank (e.g. soft exoskeletons). However, the reviewed robotic assessments are still performed under non-functional and static or passive conditions. Therefore, further development is necessary to be able to assess joint impedance during a dynamic task such as walking. A method to estimate joint impedance during gait is to use musculoskeletal models and using optimization techniques to estimate muscle forces that are related in the model to muscle impedance [169]. An alternative method is to apply time-varying system identification algorithms to estimate the changing impedance of the human knee over the gait cycle [170]. The ensemble-based correlation technique averages over repetitions instead as over the time cycle [171]. Averaging over repetitions and over time within a short data segment within repetition can also be combined [172] with the advantage that less repetitions are needed. A testing platform consisting of a knee perturbator has been built in order to deliver velocity perturbations during walking and record reaction torques, with the aim of determining the knee impedance using system identification techniques [173]. The ensemble-based correlation technique has also been applied to estimate the modulation of the ankle impedance from the end of the stance phase to heel contact with MIT’s AnkleBot [49]. However, comparing the estimated knee impedance of the ensemble-based correlation method with the model-based method [158] shows order of magnitude differences in the estimated knee impedance. Hence, more research is needed to reliably estimate the impedance of multiple joints during gait.

## Walking function/Gait pattern

### Definition of the measure

Walking can be defined as a repetitious sequence of limb motions that move the body forward while simultaneously maintaining stance stability [174]. Gait refers to the manner or style of walking [175]. Gait is composed by a cyclic series of motion patterns performed by the hip, knee and ankle. The gait cycle can be divided in phases, the main ones being swing and stance [174, 176, 177]. Walking can be described according to different domains: *i*) the capacity of performing activities related to walking (e.g. walking without assistance, sit-to-stand); *ii*) the spatio-temporal characteristics (e.g. speed, step length, cadence, stance/swing ratio); *iii*) the “quality” of gait pattern, which concerns the ability to coordinate lower-limb segments and joints (e.g., simultaneous coordination of hip and knee angles) [178].

### Clinical assessment and open issues

In clinical practice, walking is mainly assessed by examining the spatio-temporal characteristics and the capacity of performing walking-related tasks. Like the other assessments discussed in this paper, measuring walking is also influenced by time constraints. Therefore, measures that are relatively easy and fast to administer are normally chosen.

Among these, the capacity of performing functional walking activities is commonly assessed using ordinal-based clinical scores. These tests have a low administrative burden and they can be useful to grossly categorize the patients according to their walking capacity, but they are not sensitive enough to detect small improvements in locomotion [179]. The Walking Index for Spinal Cord Injury (WISCI II), for example, assigns a score between 0 and 20 based on the amount of assistance required for walking (e.g., walking with one/two crutches). Consistent floor and ceiling effects and a low responsiveness were reported [180]. Moreover, the different levels are unevenly spaced, meaning that a change of 1 point in the score has a different relevance depending on the position along the scale [179]. Several other activity-based tests were developed (e.g., Functional Ambulation Category (FAC), Dynamic Gait Index (DGI)) to assess walking function but, although very useful for gaining information on the overall walking process, they are unable to provide any detailed information on the way it is realized.

Time-based tests are often performed, since they provide quantitative measures and have shown substantial inter- and intra-rater reliability [181]. For example, in the 10-m-Walking-Test (10MWT) a stopwatch is used to measure the time required to walk 10 m [182]. Thus, the test provides a measure of short-duration walking speed and it has substantial correlation with other time-based walking tests and with other walking-related functions like muscle strength of the lower limb (Table 3) [180]. However, the information obtained with this test is limited to gait speed, which, although normally used as a surrogate measure for gait quality [183], is not able to provide information on complex alterations of walking (e.g., compensatory strategies) [184]. 10MWT and other time-based walking tests (e.g., Time-Up-and-Go, 6-min-Walking-Test) present floor and ceiling effects since non-ambulatory subjects score 0 and mildly impaired patients could walk longer distances at the same speed [180]. Other spatio-temporal parameters can be obtained using more sophisticated instruments like IMUs [185–187] and pressure mats [188]. Heel strike events can be detected from an IMU placed at the lower back [189]. A more detailed step segmentation is possible if the IMUs are placed directly on the feet [190, 191]. Parameters such as step duration, step length and swing/stance time ratio can provide important additional information on gait impairments and on the progresses during recovery. For example, there is evidence that step variability (i.e. variability in stride time, stride length and gait speed) is altered in patients with neurodegenerative diseases [192]. In stroke patients, asymmetry in right and left step time and altered stance/swing time ratio were reported using IMUs [193–195]. IMU-based systems are not yet widely integrated in clinical practice, even if new systems are now commercialized (e.g. McRoberts DynaPort [196], GaitUp [197]).

It is important to evaluate the patient’s gait pattern to understand whether the person is using compensatory strategies. These strategies might indeed not be visible in the spatio-temporal gait characteristics, which can be similar to physiological ones even in presence of an aberrant muscle activity [198]. This is especially important in longitudinal studies which aim at demonstrating whether improvements in walking speed are attained either by using compensatory strategies or by restoration of the pre-morbid gait patterns [4]. By using measurements able to capture the quality of the gait pattern it is possible to discriminate between the two different recovery strategies - compensation or restoration of physiological gait. However, at present the quality of the gait pattern can only be accurately assessed using a motion tracking system and force plates. This instrumented gait analysis provides an accurate measure of joint angles, moments and powers but requires a costly equipment and a long administration time.

A major issue related to walking assessment is that non-ambulatory subjects are often assigned the lowest score in every test (e.g. 0 m/s in the 10MWT, 0 score in the WISCI II), irrespective of their residual lower limb functions. These subjects are therefore excluded from it because most of the scales’ floor effect. It would be possible to assess non-ambulatory subjects indirectly by measuring other variables that correlate with walking ability, like muscle strength or balance. However, these tests are performed usually while sitting or lying, in contexts very dissimilar to walking.

### State of the art in rehabilitation robotics

Driven gait robots for treadmill walking and exoskeletons for overground assistance can be used to record joint kinematics while walking in order to obtain information on the quality of the gait pattern. Robotic exoskeletons equipped with angular position sensors have been utilized to record joint kinematics during treadmill or overground walking [36, 42, 199–201].

The Lokomat and the LOPES have been used to measure hip and knee angles in various studies, where the reduced impedance of the joints allowed the subjects to impose their own gait pattern. The joints’ kinematics was evaluated mainly by comparing it with a reference angular trajectory: e.g. timing error within a tunnel around the desired spatial path [202] or spatial tracking error [203]. A method to assess retraining in stroke patients based on the areal difference between a healthy reference and the patient’s trajectory during the swing phase was implemented in the ALEX gait trainer [36]. However, at present, robotic gait trainers might not be the most suitable devices for performing an assessment equivalent to camera-based gait analysis, due to the influence that their mechanical constraints have on the gait pattern. Wearable and lightweight devices that do not hinder human movements are required for this purpose. Particularly suitable for this condition would be the soft lower limb exoskeletons (“exosuits”) that have been recently developed to improve human-robot interaction and to allow a more natural walking pattern [61, 204, 205]. In an active soft orthotic ankle device two IMUs placed on the shank and on the foot are used to compute the ankle joint angle [204]. Alternatively, strain sensors embedded in the suit spanning over a joint are used [61]. Although this is a promising approach for measurements in dynamic conditions, the sensing accuracy is at present not high enough for accurate measurements, due to relative movements between the suit and the skin of the subject. Moreover, sensor calibration is required every time a user wears the suit.

The robotic assistance required for walking has been proposed as an alternative method for assessing the walking function. For example, adaptive algorithms automatically adjust the support provided by the device based on the patient’s ability to follow a predefined trajectory or to perform a specific task (e.g. foot clearance) [200, 201, 206, 207]. The algorithms update a control parameter *K* (usually the impedance of the joints and the unloading of the body weight) at each walking step *s* based on a forgetting factor *γ* < 1 and on the weighted error *g* ⋅ *e* calculated in the previous step:

1 $$ {K}_{s+1}=\gamma \cdot {K}_s+g\cdot {e}_s $$

After a certain number of steps, the parameter *K* converges to a value that can be retained as a measure of the subject’s impairment [208]. For example, an overall score can be obtained summing the torques required at each joint averaged during the last 10 steps [206].

### Future developments in rehabilitation robotics

Robotic gait trainers and exoskeletons for overground assistance can be easily instrumented to provide kinematic and kinetic data that can be used to derive metrics useful for assessing the gait pattern and the walking function. Since these devices enable non-ambulatory patients to walk in a safe and functional manner, they allow the assessment of these category of subjects, limiting unwanted floor effects of the tests. Although these systems are expensive, they are already used in many clinical centers worldwide for providing gait training. In these contexts, subjects can be tested during gait training, requiring no or little additional time. Repeatable assessment procedures can be programmed in order to standardize the testing conditions (e.g., speed, unloading of body weight). Accurate measurements of the gait pattern can be obtained if the effects of the device dynamics (i.e. weight, inertia) are minimized. Moreover, the exoskeletons should have enough degrees of freedom to avoid constraining physiological walking movements. The compliant fixation of the patient’s leg to the orthosis could lead to measurement inaccuracy and errors [209], therefore standardized procedures need to be established in order to make the patient’s setup in the device as independent as possible of the operator. When the transparency of the device is guaranteed by hardware design (e.g. soft exoskeletons) or by software compensation [52, 53], the robot can be used for measuring joint kinematics or spatio-temporal gait parameters. When this condition is not met or when the subject is too impaired to be able to walk without the support of the device, other assessment methods must be used. It would be misleading, in fact, to measure standard gait parameters in a robotic gait trainer that affects the patient’s walking pattern. To address this problem, new outcome measures can be proposed. For example, the amount of support (i.e. joint impedance or unloading of the body weight) required to achieve a functional walking pattern can be used as an indicator of the subject’s impairment. Further studies in this direction are needed to establish the concurrent validity of this outcome measure with existing clinical scores. It can be hypothesized that a correlation with clinical scores that address the amount of support required for walking (e.g. WISCI II, FAC) exists. Moreover, if the algorithm adapts the support of the device to the particular needs of the single gait phases, it would be also possible to identify specific impairments localized within the gait cycle [206]. However, the results of this method depend on the performance metric used. If a measure of the deviation from a reference trajectory is applied, the resulting support will depend also on the similarity between the prescribed trajectory and the patient’s individual gait pattern. A dead band around the reference trajectory, as in [206], could partially address this problem.

## Discussion and conclusion

In this review we have discussed how novel robot-aided functional assessments can address the current issues related to the evaluation of the health-related status of a patient in clinical practice. Although essential for maximizing the individual therapy outcomes, the use of assessment methods in routine practice is at present insufficient. Among the reasons that contribute to this dearth, poor quality of the existing assessment scores and high administrative burden have been identified [1]. In the different sections of this review we have highlighted additional issues in current clinical assessments specific for different lower limb functions. We have explained how robotic devices for rehabilitation have the potential to solve these issues by providing high quality assessments (i.e. objective, reliable and valid) and by integrating the assessment procedure in a training program. Based on the existing shortcomings and on the possibilities offered by robotic technologies, we have proposed solutions and recommendations for the development of novel robot-aided assessment tools. The quality of the assessment methods must be determined by studying their psychometric properties, as discussed in the section *Assessments validation*. We believe that the increasing use of robots for rehabilitation is not only beneficial for the therapy outcome, but also represents a huge opportunity for improving the assessment quality and increasing their frequency of administration. Indeed, robotic devices can be equipped with sensors for recording data useful for developing quantitative and objective assessment metrics. Secondly, robots can potentially assure the standardized execution of the assessment procedure, which is essential for reducing the measurement error and increasing the reliability. Moreover, robot-based assessments can reach higher inter-rater and intra-rater reliability if the robotic device is designed to limit fixation errors and to reduce inter-operator differences. Cuffs positioning, misalignments and different tightening of the fixation to the patient’s limbs may have a huge impact on the reliability of the assessment outcomes. User-friendly and ergonomic robotic device, along with a rigorous training of the operators may contribute to solve this problem.

A known issue of the current assessments used in clinical practice is their administrative burden (mainly time-wise) that limits the frequency at which they are administered. Assessments executed with rehabilitation robotic devices can be performed during the therapy session, measuring relevant parameters directly during the training, while the patient is using the device. Robotic assessments are able not only to complement existing clinical measurements, but also to enlarge the measurable range of an impairment: because of the quantifiable assistance that robots can provide, robotic assessments can be administered even if the patient is not able to perform the movement without support [6]. Moreover, measurements that have been only subjectively addressed before (e.g., proprioception) can now be targeted by instrumented tests. New variables that were not readily accessible before (e.g., smoothness, joint coupling) become available. Further research on neurophysiological mechanisms must be encouraged to determine how these variables relate to sensorimotor functions and whether they can provide information on recovery [6]. The increased sensitivity and the reduced measurement error of the robot-based assessments can be of utmost importance when the outcome measures are used in a clinical trial aimed at demonstrating the efficacy of a new therapy. Often little can be concluded because the effects of the therapy under study are masked by high inter-subject variability or they are not captured by conventional clinical assessments [5]. Assessments able to distinguish the contribution of restoration of physiological patterns and the effect of compensatory mechanisms to the recovery will help to orient future therapeutic approaches [4]. Not least, more sensitive measurements could also contribute to increasing the motivation of the patients, when even a slight improvement can be documented.

Before starting a research study aiming at developing a new assessment method, researchers must consider several issues. First of all, an inter-disciplinary approach involving research institutes, clinical facilities and medical device manufacturers is encouraged in all the phases of development: researchers must take into account the clinical relevance of the proposed measure (is the information provided by the measure useful for adjusting the therapy?), the interpretability of the outcome parameter (what is its physiological meaning?), the feasibility of the method for both its use in clinical practice (is it safe? Are its administrative and respondent burdens reasonable?) and the manufacturability and large-scale implementation. If these steps are missing, the risk is that the assessment method will never be routinely used in the clinical practice. On the other side, it is also important that researchers go “beyond” the limits of established clinical tests by developing new and independent standards based on robotic measurements. The final aim of robotic assessments, indeed, should not be to reproduce existing clinical scales that, even if widely accepted in the clinical practice, are not comparable by their nature to instrumented and robot-based assessments [6]. Lastly, when developing assessment metrics of a same variable for different lower extremities devices, researchers should try to make the results independent of the platform on which they are obtained. In this way the same metric could be implemented in different devices and results from several studies could be compared. Even if the dynamics of the device will most likely influence some of the assessment metrics, comparative measures can be used (e.g. normalizing patient’s data against healthy normative data recorded in the same device).

A crucial requirement for the acceptance of a new assessment method in the rehabilitation community is its clinical validation: reliability must be assessed with an adequate sample size and validity should be established either by comparing the score with a gold-standard - if it exists - (concurrent validity) or by studying the relationship of the new assessment score with the underlying constructs of interest (construct validity). Guidelines for the validation of new robot-based assessments should be developed to help the researchers to define adequate clinical validation studies and to use the correct statistical tools. Moreover, it is necessary to develop indications for interpreting the different scoring systems: clinicians must be able to identify whether a change in score is clinically significant or it is due to measurement error [3].

We think that robotic assessments represent a challenging “green field” where researchers have the possibility – and the urgency – to develop methods that will have a strong impact on rehabilitation outcomes. Better assessments of lower extremities functions will allow the clinicians to prescribe therapeutic and rehabilitation plans that optimize the individual recovery while minimizing unnecessary effort and costs [210]. We believe, therefore, that research for developing valid, reliable and responsive assessment methods is strongly needed for clinical practice, for studies on new therapies and, overall, for improving the rehabilitation outcome and decreasing the time of recovery.

## Abbreviations

10MWT, 10-m-walking-test; 1-RM, one repetition maximum; 6MWT, 6-min-walking-test; AB, able-bodied; CI, confidence interval; CP, cerebral palsy; CPM, continuous passive motion; DF, dorsiflexion; DGI, dynamic gait index; DOF, degree of freedom; EMG, electromyography; FAC, functional ambulation category; HHD, hand-held dynamometer; ICC, intra-class correlation coefficient; ICF, international classification of function, disability and health; JPR, joint position reproduction; LOA, limits of agreement; MAS, modified Ashworth scale; MCID, minimum clinically important difference; MDC, minimum detectable change; MMT, manual muscle test; MVC, maximum voluntary contraction; OA, osteoarthritis; PF, plantarflexion; ROM, range of motion; SD, standard deviation; SEM, standard error of measurement; TTDPM, time to detection of passive motion; TUG, timed-up-and-go; WISCI II, walking index for spinal cord injury (2nd version)

## Footnotes

1 The terminology used in different publications is not always consistent. In the work of Kearny and colleagues [136] the intrinsic component includes the passive properties of the joint and the properties of the active muscle fibers.
